# Arrestin-Mediated Endocytosis of Yeast Plasma Membrane Transporters

**DOI:** 10.1111/j.1600-0854.2009.00990.x

**Published:** 2009-12

**Authors:** Elina Nikko, Hugh R B Pelham

**Affiliations:** MRC Laboratory of Molecular BiologyHills Road, Cambridge CB2 0QH, UK

**Keywords:** arrestin, endocytosis, transporter, ubiquitin, ubiquitin ligase, yeast

## Abstract

Many plasma membrane transporters in yeast are endocytosed in response to excess substrate or certain stresses and degraded in the vacuole. Endocytosis invariably requires ubiquitination by the HECT domain ligase Rsp5. In the cases of the manganese transporter Smf1 and the amino acid transporters Can1, Lyp1 and Mup1 it has been shown that ubiquitination is mediated by arrestin-like adaptor proteins that bind to Rsp5 and recognize specific transporters. As yeast contains a large family of arrestins, this has been suggested as a general model for transporter regulation; however, analysis is complicated by redundancy amongst the arrestins. We have tested this model by removing all the arrestins and examining the requirements for endocytosis of four more transporters, Itr1 (inositol), Hxt6 (glucose), Fur4 (uracil) and Tat2 (tryptophan). This reveals functions for the arrestins Art5/Ygr068c and Art4/Rod1, and additional roles for Art1/Ldb19, Art2/Ecm21 and Art8/Csr2. It also reveals functional redundancy between arrestins and the arrestin-like adaptors Bul1 and Bul2. In addition, we show that delivery to the vacuole often requires multiple additional ubiquitin ligases or adaptors, including the RING domain ligase Pib1, and the adaptors Bsd2, Ear1 and Ssh4, some acting redundantly. We discuss the similarities and differences in the requirements for regulation of different transporters.

Many yeast plasma membrane transporters are removed from the cell surface in response to excess substrate, nutrients or stress. It is well established that their endocytosis and subsequent vacuolar degradation are controlled by ubiquitination, and in every case tested, ubiquitination requires the HECT domain E3 ligase Rsp5 (1).

How a single ubiquitin ligase recognizes a wide variety of substrates is now beginning to be understood. Rsp5 contains three WW domains, which recognize PY motifs with the typical sequence PPXY or LPXY. Several adaptor proteins containing such motifs have been shown to facilitate the ubiquitination of particular proteins or sets of proteins (reviewed in (2)). These adaptors include the membrane proteins Bsd2 (3), Tre1/2 (4), Ear1, Ssh4 (5) and possibly Rcr1/2 (6,7), as well as the soluble proteins Bul1 and Bul2 (8–10). Most recently, members of a family of yeast arrestin-like proteins have been shown to mediate the ubiquitination and endocytosis of the metal transporter Smf1 and the amino acid transporters Can1, Mup1 and Lyp1 in response to stress or the corresponding amino acids (11,12). These arrestins contain PY motifs, and bind to and are ubiquitinated by Rsp5. In the case of Smf1, recognition by the arrestin requires phosphorylation of the transporter, reminiscent of the phosphorylation-dependent interaction of mammalian arrestins with G-protein coupled receptors (12). In the case of the lysine permease Lyp1, it was found that endocytosis in response to stress (cycloheximide treatment) required a different arrestin (Art2/Ecm21) compared with lysine-induced endocytosis (Art1/Ldb19) (11), and Art2 is also one of the arrestins that mediates stress-induced endocytosis of Smf1.

Although these studies suggested a general model in which different arrestins recognize different transporters, or the same transporter in response to different stimuli, the results were limited mainly to two of the nine reported arrestins (Art1/Ldb19 and Art2/Ecm21) and to the four transporters mentioned above. Several other permeases appeared unaffected in arrestin mutant strains, possibly because of functional redundancy of the arrestins. We therefore sought to extend the findings to other proteins, to test the generality of the model and see whether a single mechanism can account for the behaviour of all regulated transporters.

Here we identify adaptors required for regulated degradation of the inositol permease Itr1, hexose transporter Hxt6, uracil permease Fur4, and tryptophan transporter Tat2. We show that Bul1 and Bul2 have functions related to those of the arrestins and may share structural features with them. We also show that the Rsp5 adaptor Bsd2 and the RING domain ligase Pib1 provide an additional redundant function required for several transporters to negotiate the multivesicular body pathway. The results reveal common themes but also considerable variation in the requirements for individual proteins to be endocytosed to the vacuole.

## Results

### Arrestin-like proteins in yeast

Our previous studies suggested considerable functional redundancy amongst the yeast arrestins. Our strategy therefore was to eliminate all of them, test the effect on a given transporter, then add back plasmids encoding single arrestins to see which were best at restoring endocytosis. This strategy required all of the relevant arrestins to be identified.

Yeast contains eight proteins that have clear arrestin sequence signatures and show readily detectable homology to each other, namely Art2/Ecm21, Art8/Csr2, Art4/Rod1, Art7/Rog3, Art6/Aly1, Art3/Aly2, Art5/Ygr068c and Art9/Rim8 (11,12). In addition, a much more distantly related protein, Art1/Ldb19, which is still clearly identifiable as a member of the arrestin family, was discovered by functional screening (11). Most of these proteins have been shown to bind to Rsp5 and be ubiquitinated by it (13,14), the exception being Art9/Rim8. Rim8 lacks canonical PPXY elements and has a unique role in pH regulation (15), and thus may have a function distinct from that of the others.

**Figure 1:**
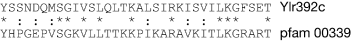
Alignment of residues 18–51 of Ylr322c with part of the pfam consensus sequence for the arrestin N-terminal domain, with identities and similarities indicated The full alignment is detectable by automated domain searches, but has a relatively high *E*-value of 0.1.

We identified an additional protein, Ylr392c, which also shows a match to the pfam arrestin_N domain (Figure 1) yet is distantly related both to Art1 and to the other members of the family. It has PY motifs, and has been shown to be an Rsp5 substrate (14). On the basis of these properties, we propose the name Art10 for this protein. Table 1 lists the various gene names and relationships. For simplicity, in this paper we use the Art nomenclature (Arrestins Related to Transport) proposed by Lin et al. (11).

**Table 1:** Names of yeast arrestins

Art	Systematic	Other	Closest relative
Art1	yor322c	Ldb19	
Art2	ybl101c	Ecm21	Art8
Art3	yjl084c	Aly2	Art6
Art4	yor018w	Rod1	Art7
Art5	ygr068c		Art9
Art6	ykr021w	Aly1	Art3
Art7	yfr022w	Rog3	Art4
Art8	ypr030w	Csr2	Art2
Art9	ygl045w	Rim8	Art5
Art10	ylr392c		

We constructed a yeast strain in which all 10 arrestins were deleted. Although viable, this strain grew more slowly than wild-type. This was at least partly because of the loss of Rim8, which is required for robust growth under a variety of conditions. Because Rim8 seems to have a distinct function, for subsequent endocytic studies we primarily used a *9-arrestin* mutant, lacking all the PY-containing arrestins, which was healthier. To allow studies of the endocytosis of the metal transporter Smf1 this strain also lacked Bsd2, an adaptor that in normal medium causes newly synthesized Smf1 to be diverted from the Golgi to the vacuole. We refer to this strain as the *9-arrestin* mutant.

### Endocytosis of Itr1 is mediated by Art5

**Figure 2:**
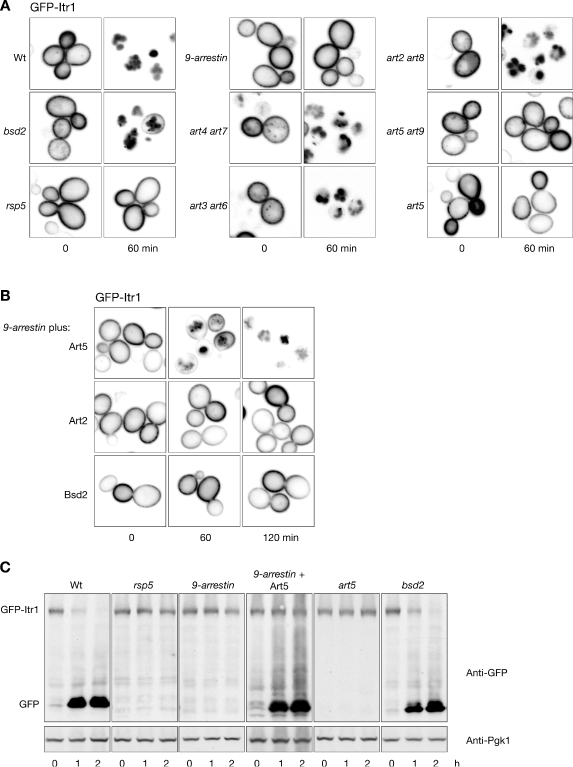
Itr1 degradation requires Art5 A) Confocal images of GFP-Itr1 in the indicated strains. These were grown in the absence of inositol, GFP-Itr1 synthesis induced with galactose, induction terminated with glucose, then inositol added and samples examined 0 or 60 min after this. Images are shown inverted for clarity. B) GFP-Itr1 in the *9-arrestin* mutant with the indicated proteins expressed from plasmids, imaged as in A at the indicated times after the addition of inositol. C) Immunoblots of GFP-Itr1 in the indicated strains. Pgk1 was detected on the same blots as a loading control.

The inositol transporter Itr1 is endocytosed in response to exogenous inositol (16). It is found at the cell surface in inositol-depleted medium, as shown in Figure 2A for green fluorescent protein-ltr1 (GFP-Itr1) transiently expressed from a GAL1 promoter. Addition of inositol triggered its endocytosis and delivery to the vacuole (Figure 2A). Unlike some other transporters, stressing the cells by cycloheximide or cadmium treatment was not sufficient to induce internalization of Itr1 (not shown).

As expected, internalization was defective in an *rsp5* mutant, and also in the *9-arrestin* mutant strain (Figure 2A), though not in the *bsd2* parental strain. We then tested a series of strains lacking related pairs of arrestins, and found that endocytosis of Itr1 was affected only in *art5 art9*. The same was true in a single *art5* mutant. Finally, we added plasmids expressing each of the nine individual arrestins, and Bsd2, back to the *9-arrestin* strain, and found that Art5 restored trafficking of Itr1 (Figure 2A) whereas none of the others did (Figure 2B and data not shown).

These results were confirmed by monitoring the appearance of free GFP on immunoblots–because GFP is resistant to vacuolar proteases, it accumulates after GFP-Itr1 is cleaved in the vacuole (Figure 2C). It is also detected much more efficiently than the tagged transporters from which it is derived, possibly because it transfers to the blot more easily. Plasmid-expressed Art5 restored GFP-Itr1 cleavage, though not quite to normal levels as indicated by residual intact protein (Figure 2C). This may reflect less efficient expression from the plasmid construct. Nevertheless, the results show clearly that Art5 is necessary for the inositol-induced internalization of Itr1, and is the only arrestin required for this. This is the first function identified for this arrestin.

### Endocytosis of Hxt6 involves Art4 and Art8

**Figure 3:**
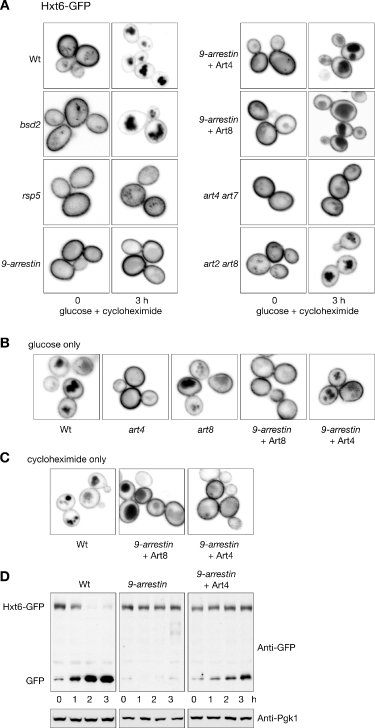
Requirements for Hxt6 degradation A) Hxt6-GFP expressed from the TPI promoter in cells of the indicated strains, grown in raffinose then transferred to glucose for the indicated times, with cycloheximide added at the same time as the glucose. B) As A, but with no cycloheximide. Cells were in glucose for 1–2 h. C) As A, but only cycloheximide added, for 2 h. D) Immunoblots of cells treated with both glucose and cycloheximide, as indicated.

We used a similar approach with the high affinity hexose transporter Hxt6, which is degraded in response to high glucose (17). Because it is controlled by sugars we avoided use of a galactose-inducible promoter and instead used the constitutive TPI promoter for expression. GFP-tagged Hxt6 was mainly at the cell surface when cells were grown in raffinose, but was slowly transported to the vacuole upon shift to glucose, together with the addition of cycloheximide to block further synthesis of the transporter (Figure 3A).

Figure 3A shows that Hxt6 trafficking was normal in a *bsd2* strain, but blocked in both *rsp5* cells and in the *9-arrestin* mutant. Adding back plasmid-borne genes showed at least partial rescue by Art4, as well as by Art8 (Figure 3A), but by no other single arrestin nor by Bsd2 (not shown). Analysis of double arrestin mutants confirmed the importance of Art4: *art4 art7* cells did not internalize Hxt6 efficiently. In contrast, removal of the Art2 Art8 pair which show redundancy for Smf1 regulation (12), did not have any significant effect (Figure 3A).

A potential complication in these experiments was that cycloheximide, added to stop new synthesis of Hxt6, is known to induce internalization of some transporters, and thus may provide a signal in addition to the effects of glucose addition. By using glucose treatment alone, we could confirm that only Art4 is essential for glucose regulation: *art4* cells were defective, whereas *art8* cells were not; in addition, Art8 could not complement the *9-arrestin* mutant in this assay whereas Art4 could to at least some extent (Figure 3B). When cycloheximide treatment alone was used, Art8 was able to complement, but Art4 had little activity (Figure 3C). Thus, in normal cells the bulk of Hxt6 regulation by glucose appears to be mediated by Art4, whereas stress-induced internalization is mediated mainly by Art8.

The key features of Hxt6 regulation were confirmed by immunoblotting, which showed complete inhibition of degradation in the *9-arrestin* mutant, with partial rescue by Art4 (Figure 3D). In these experiments, cells were treated with both glucose and cycloheximide, as the single treatments gave much weaker effects. In general, expression of a single arrestin never restored endocytosis to wild-type levels, suggesting that multiple arrestins contribute in an additive fashion to Hxt6 trafficking, as we have previously observed for Smf1 (12).

### Multiple adaptors control Fur4 endocytosis

We next examined the uracil permease Fur4. This protein has been extensively studied, and it has been shown that the addition of uracil leads to rapid internalization from the cell surface (18). Fur4 is also very sensitive to cycloheximide stress, which similarly results in internalization and vacuolar degradation (19).

**Figure 4:**
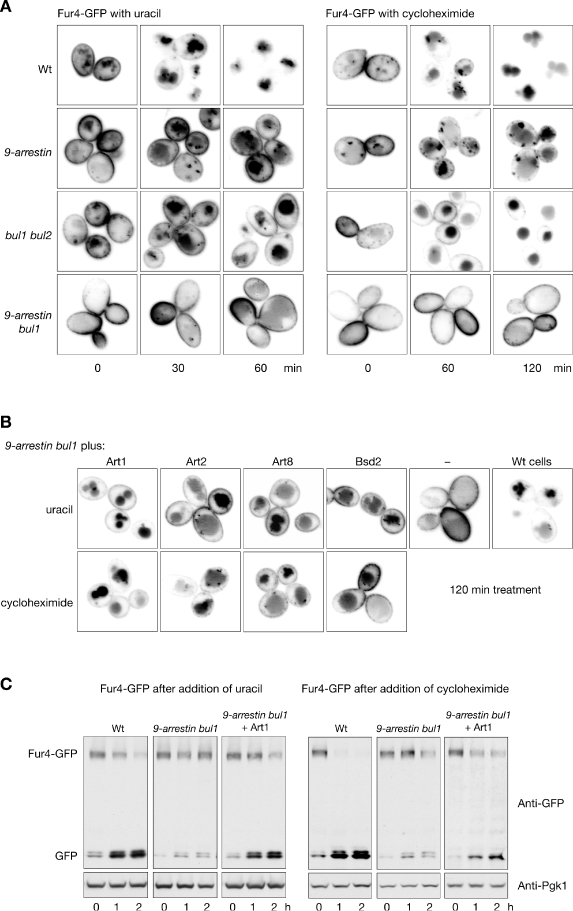
Requirements for Fur4 degradation A) Images of Fur4-GFP in various strains after transient galactose induction, at the indicated times after addition of uracil or cycloheximide. B) Fur4-GFP in the indicated strains after treatment with uracil or cycloheximide for 2 h. Note that the *9-arrestin* strain lacks *BSD2*. C) Immunoblots of Fur4-GFP as indicated.

We were surprised to find that uracil-induced internalization of Fur4 was not completely blocked in the *9-arrestin* mutant (Figure 4A) or even in a strain lacking all 10 arrestins (not shown). This suggested that there was an alternative adaptor capable of recognizing this protein. As Bul1 and its relative Bul2 are known to bind Rsp5 and have been implicated in the regulation of the general amino acid permease Gap1 (9,20), we also tested a *bul1 bul2* double mutant. These cells were still capable of transporting Fur4 to the vacuole upon uracil addition, but did so slightly less efficiently than wild-type cells. In both the *arrestin* and *bul* mutants there was some accumulation of permease in punctate endosome-like structures, indicating that inefficient ubiquitination may impair not only internalization but also later steps in the endocytic pathway. This is discussed further in the following text.

The incomplete blocks suggested redundancy, and to test this we deleted *BUL1* and *BUL2* from the *9-arrestin* mutant. The resultant cells grew very slowly (Table 2), indicating a synthetic interaction between the *BUL* genes and one or more of the arrestins, which in turn suggest that they do share some essential function. This strain grew so poorly that we could not easily characterize it, but a strain lacking the nine arrestins and Bul1 grew better (Table 2), and in this strain Fur4 endocytosis was much more strongly blocked than in either the *bul1 bul2* or the *9-arrestin* strain (Figure 4A). In addition, we noticed that the cells tended to be oval in shape. Similar results were obtained when cycloheximide was used to induce Fur4 internalization (Figure 4A).

**Table 2:** Growth rate of mutants

Strain	Doubling time (min)
wt	108
*bsd2*	105
*9-arrestin*	137
*bul1/2*	146
*9-arrestin bul1/2*	329
*9-arrestin bul1*	159

**Figure 5:**
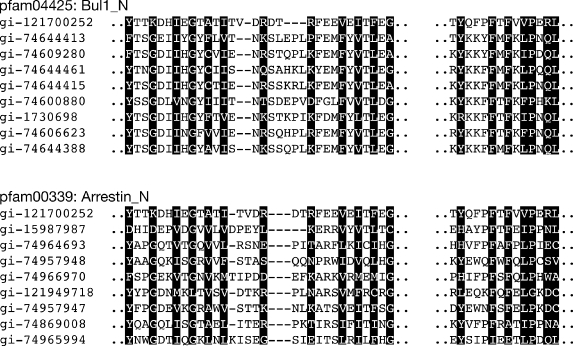
Alignment of parts of the Bul1 and arrestin conserved regions, as identified by pfam The top line in each case consists of residues 39–68 and 112–125 from a single *Aspergillus clavatus* protein. Residues that are similar in both the Bul1 and arrestin families are highlighted. The Bul1 sequences are all fungal; the arrestins are from a wide variety of species, and show considerable divergence.

The finding that Bul1 might perform a similar function to that of the arrestins prompted us to examine the Bul1 protein sequence for arrestin motifs. Like the arrestins, the Bul proteins are predicted to be largely beta sheet proteins, repeated in two tandem domains. Strikingly, psi-Blast homology searches (21) identify similarities between Bul1 and some fungal arrestin-like proteins. We identified one protein from *Aspergillus clavatus* which contained similarity to both the Bul1_N and Arrestin_N domains, as defined by pfam (22). Figure 5 shows that short regions of similarity can indeed be detected between multiple sequence alignments representing these domains. This suggests that the Bul proteins may represent a fungal-specific distant variant of the arrestin family, with at least some functions in common with the arrestins.

To identify which particular arrestin mediates Fur4 endocytosis, we complemented the *9-arrestin bul1* strain with each arrestin, and found that Art1 and Art2 were most effective, whilst Art8 showed weaker activity (Figure 4B). It has been reported that Art2 mediates stress-induced internalization of Lyp1, whereas Art1 is required specifically for lysine-mediated endocytosis of the same transporter (11). However, in the case of Fur4 we did not see a clear preference; both arrestins could complement cycloheximide as well as uracil-mediated internalization (Figure 4B).

Surprisingly, we also observed partial rescue of vacuolar delivery when we complemented the *9-arrestin bul1* mutant with Bsd2, which is also absent from this strain (Figure 4B). This is striking because Bsd2 is a membrane protein that itself is ubiquitinated and normally travels directly from the Golgi to endosomes (3), and thus it would not be expected to aid endocytosis. However, it is possible that some Bsd2 transiently reaches the plasma membrane, or that Fur4 undergoes some cycling from the plasma membrane to endosomes and back in the *9-arrestin bul1* strain, and thus contacts Bsd2 in endosomes.

Immunoblotting of Fur4-GFP supported these results. Appearance of free (vacuolar) GFP in response to either uracil or cycloheximide was reduced in the *9-arrestin bul1* strain, and this was partially restored when Art1 was expressed. It appears that Fur4 is regulated by a number of different adaptors including Art1, Art2, Art8, Bul1, Bul2 and Bsd2, and it may be that no one protein confers full activity.

### Requirements for Tat2 endocytosis

**Figure 6:**
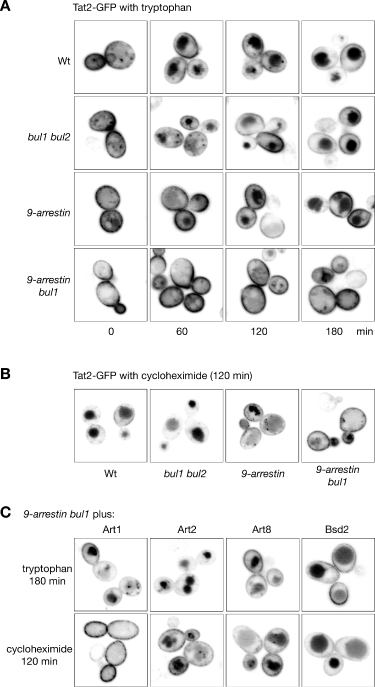
Tat2 endocytosis A) Images of Tat2-GFP in the indicated strains, induced transiently with galactose then exposed to tryptophan for the indicated times. B) As A, but endocytosis induced by treatment with cycloheximide for 2 h. C) Tat2-GFP in the *9-arrestin* mutant expressing individual proteins from plasmids, as indicated, after treatment with tryptophan or cycloheximide.

We found that endocytosis of the tryptophan transporter Tat2 had many similarities with that of Fur4, and the results are summarized in Figure 6. Tat2 is targeted to the vacuole when cells are grown in high tryptophan medium (23), and we observed that pre-synthesized Tat2-GFP was slowly endocytosed to the vacuole upon tryptophan addition. As with Fur4, endocytosis could only be blocked efficiently if *BUL1* was deleted in addition to the arrestins (Figure 6A). Cycloheximide also induced endocytosis, with similar requirements (Figure 6B). With tryptophan, Art1 and Art2 restored activity quite well, and Art8 and Bsd2 partially. Strikingly, however, Art1 did not restore the cycloheximide response, though the other proteins did (Figure 6C). This contrasts with Fur4, for which Art1 restored both stress- and uracil-induced degradation.

### Redundant roles for Bsd2 and the RING domain ligase Pib1

The fact that efficient degradation of Fur4 appeared to require both an arrestin and the endosomal protein Bsd2 raised the question of whether in general multiple ubiquitination events are required for a plasma membrane protein to reach the vacuole, and how they are mediated. There are clear suggestions that additional steps are needed, and evidence that a related pair of endosomal Rsp5 adaptors, Ear1 and Ssh4, are required for the entry of multiple proteins into the multivesicular body pathway, including Fur4 (5). Of the proteins tested in the previous publication, only the metal transporter Smf1 did not require these proteins, possibly because Smf1 is a good substrate for Bsd2-mediated ubiquitination in the Golgi or endosomes. However, we have shown that in stressed cells Smf1 is recognized by Art2/Ecm21 and Art8/Csr2 at the cell surface, and can be endocytosed to the vacuole in the absence of Bsd2 (12). We therefore reinvestigated whether arrestins are sufficient for vacuolar delivery under these conditions, or whether additional ubiquitin ligases or adaptors are required.

**Figure 7:**
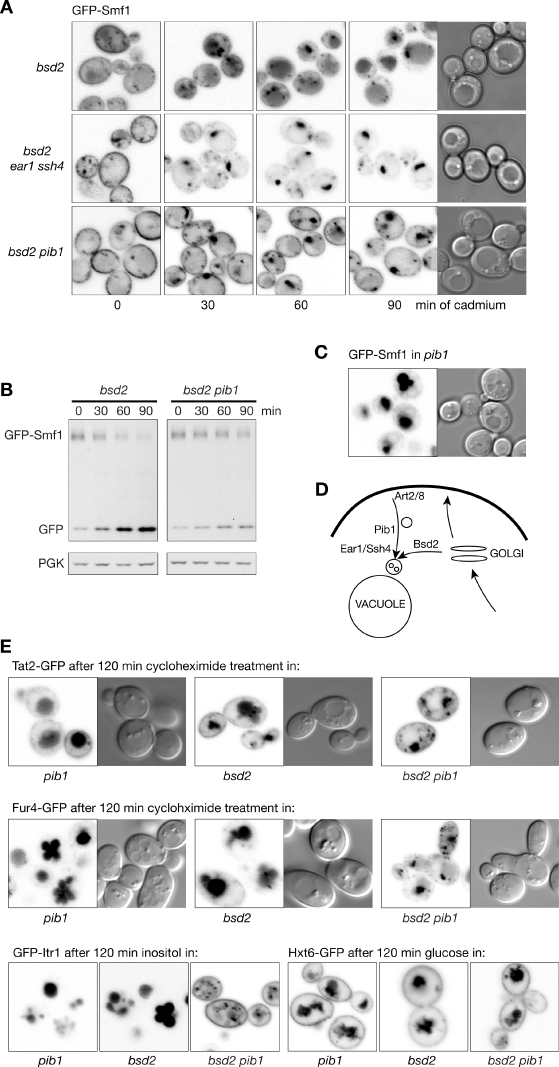
Role of Pib1 and Bsd2 A) GFP-Smf1 in the indicated strains, at various times after addition of cadmium chloride to induce Smf1 endocytosis. Differential interference contrast (DIC) images of the final time-point are included to show that in *bsd2 ear1 ssh4* cells, and in *bsd2 pib1* cells, GFP-Smf1 accumulates not in vacuoles but in perivacuolar endosomes. B) Immunoblots showing less accumulation of free (vacuolar) GFP from GFP-Smf1 in the *bsd2 pib1* mutant compared with *bsd2*. Indicated times are after the addition of cadmium chloride. C) GFP-Smf1 in *pib1* cells grown in normal metal-replete medium. Under these conditions, Smf1 undergoes Bsd2-dependent transport from the Golgi to the vacuole. D) Summary of the routes taken by Smf1 and the postulated sites at which the various adaptors/ligases act. Note that Bsd2, Ear1 and Ssh4 are all membrane proteins, that pass through the Golgi to endosomes and into multivesicular bodies, and thus could act anywhere along this pathway. E) Images of cells expressing Tat2-GFP, Fur4-GFP, GFP-Itr1 or Hxt6-GFP, induced as in the previous figures, in the indicated strains. Hxt6 endocytosis was induced with glucose in the presence of cycloheximide. Note that GFP-labelled Tat2, Fur4 and Itr1 accumulate in vacuoles in *pib1* and *bsd2* cells, but in endosomes in *bsd2 pib1* cells.

In the absence of the Ear1-Ssh4 pair of Rsp5 adaptors, proteins such as Fur4 are reported to be endocytosed, but to accumulate in perivacuolar endosomes (5). Figure 7A shows that exactly the same phenotype occurred when Smf1 endocytosis was induced by cadmium stress in a *bsd2 ear1 ssh4* triple mutant. Thus, these adaptors are also required for Smf1 degradation when Bsd2 is absent; presumably they contribute additional ubiquitination, which can alternatively be provided by Bsd2.

In searching for other possible contributors to Smf1 ubiquitination, we tested the effects of removing the RING domain ubiquitin ligase Pib1. This ligase contains a PX domain, and is associated with PI3P-containing membranes of the endocytic pathway (24). Surprisingly, it was also required for Smf1 to reach the vacuole from the cell surface in *bsd2* mutant cells (Figure 7A). The phenotype was similar to that induced by the lack of Ear1 and Ssh4, except that there was more persistence of plasma membrane fluorescence, consistent with either slowed endocytosis or recycling of GFP-Smf1 from endosomes to the surface. The inhibition of vacuolar delivery was confirmed by the reduced production of free GFP in a *bsd2 pib1* strain relative to the *bsd2* strain (Figure 7B).

When Bsd2 is present, in normal metal-replete medium, GFP-Smf1 is targeted directly from Golgi to vacuole and GFP accumulates there (25). This process was unaffected in a *pib1* single mutant (Figure 7C). Our results can therefore be summarized as follows. Entry of Smf1 into the vacuole requires either Bsd2-dependent ubiquitination (in the Golgi and/or endosomes), or a combination of arrestin-mediated ubiquitination at the cell surface, followed by the further action of Ear1/Ssh4 and additionally Pib1 in endosomes. This is indicated schematically in Figure 7D. In addition, Golgi to vacuole diversion of Smf1 requires the Tre1/2 adaptors (4), but like Bsd2 these are dispensable for stress-induced endocytosis from the surface (data not shown).

We next asked whether Pib1 was required for vacuolar delivery of other transporters. Initial experiments suggested that it was not, but we considered the possibility that its role was masked by the activity of Bsd2. We first tested Tat2 and Fur4, as we had evidence that Bsd2 could act on these proteins. Figure 7E shows that cycloheximide treatment targeted both GFP-tagged proteins to the vacuole in a single *pib1* mutant. In *bsd2* cells vacuolar delivery was also efficient, though as with Smf1 some persistent endosomal fluorescence was sometime apparent. Strikingly, however, in the *bsd2 pib1* double mutant Tat2 was almost exclusively in endosomes after 2 h, the vacuoles being noticeable for their lack of fluorescence. Fur4 showed a generally similar effect, but vacuolar entry was only partially inhibited.

We then tested Itr1 and Hxt6, and found that Itr1 was also affected in the *bsd2 pib1* double mutant, with a phenotype similar to that of Fur4. However, Hxt6 trafficking was apparently unaffected (Figure 7E), indicating either that Bsd2 and Pib1 do not act on this transporter or simply that their action is not required for its transport to the vacuole. We conclude that, at least for four of the five proteins we have tested, Pib1 and Bsd2 promote transit of endocytosed molecules through the multivesicular body pathway in a functionally redundant manner.

## Discussion

### Arrestins and the regulation of endocytosis

The main goal of this work was to test the idea that the yeast arrestins provide the layer of specificity required for a single ubiquitin ligase, Rsp5, to mediate numerous specific endocytic events with different proteins under different conditions. Because of the known problems of redundancy, we chose to remove as many arrestins as possible and then test individual ones, expressed at normal levels, for activity under various conditions. This approach is a conservative one, designed to identify the key players. It will miss weak activity, which if contributed by several arrestins may nevertheless make a significant contribution. It is however more revealing than individual gene mutations, which often had little or no effect.

**Figure 8:**
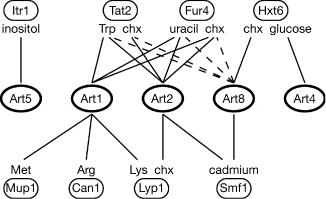
Summary of arrestin functions Arrestins are represented in the middle line, transporters in the upper line (this paper) or lower line [previous work; (11,12)]. The various substances used to induce endocytosis are shown (chx, cycloheximide). Solid lines indicate function of a particular arrestin with the indicated transporter and inducing condition. Dashed lines indicate weak activity. All activities were observed with arrestins expressed from their own promoters.

We have extended the existing analysis to four additional transporters of diverse types, and identified new functions for five of the arrestins. Figure 8 summarizes the present and previous results, and from this some patterns can be discerned. First, endocytosis induced by stress–notably cycloheximide treatment–occurs with a subset of transporters, and these always seem to respond to one or both of the related Art2/Art8 pair. Individual specificities vary–Smf1 uses both proteins, whereas Hxt6 prefers Art8 and Lyp1 prefers Art2. The simplest explanation is that stress somehow increases the activity of Art2 and Art8, and these then induce the endocytosis of any transporter that they recognize.

Another generalization is that all four amino acid transporters tested use Art1 to mediate substrate-induced endocytosis. As each responds to a different amino acid, it is unlikely that these amino acids control Art1 activity; instead, Art1 may recognize a common feature of these related transporters, perhaps binding specifically to a substrate-induced conformation or state. In contrast the sugar transporters Itr1 and Hxt6 each use a different arrestin, and it remains to be seen whether the activity of these arrestins is regulated, and whether common themes will emerge with other transporters of this family.

With Fur4 and Tat2 the situation is particularly complex. Not only are they able to use the same arrestins for both stress- and substrate-induced turnover, but in addition their degradation is also affected by the distantly related Bul proteins as well as by other adaptors. It is unclear whether there are a series of specific recognition events, or whether each of these adaptors recognize some rather common structure, such as an unfolded loop.

In this study, we have concentrated on the endocytosis of pre-synthesized protein, but it is worth noting that in the presence of their substrates both Tat2 and Fur4 can be directed from the Golgi to the vacuole before reaching the plasma membrane (23,26). Given that Bsd2 and Bul1/2 are required for the Golgi to vacuole diversion of Smf1 and Gap1 respectively (9,27), one or both of these adaptors may perform a similar function with Tat2 and Fur4. Indeed, Bul1 has been reported to be necessary for efficient targeting of Tat2 from Golgi to vacuole (23).

### Requirement for additional ubiquitination

In addition to the role of arrestins in initial internalization, this and previous work show that other adaptors or ubiquitin ligases are often required for proteins to reach the vacuole. A clear example is provided by Smf1. Trafficking of this protein from the Golgi requires the adaptors Tre1/2 and Bsd2 (4); from the surface, in the absence of Bsd2, it requires Art2/8, Ear1/Ssh4 and the RING ligase Pib1. Similarly, in addition to arrestins, Fur4 requires Bsd2 or Pib1, as well as Ear1/Ssh4 (5).

In the absence of these secondary adaptor/ligases, the transporters passage slowly through endosomal structures and accumulate in them. We suggest that this is a consequence of attached ubiquitins interacting with the early ESCRT (endosomal sorting complex required for transport) machinery (28). Evidently, however, efficient entry into multivesicular bodies requires further modification, e.g. through the formation of K63-linked ubiquitin chains (29–31), or the modification of additional lysines, or the replacement of ubiquitins that are lost. The requirement for multiple adaptors and ligases might reflect the need to create specific modifications, or to provide modification at different stages in the pathway, or simply to reach a threshold level of ubiquitination. If total levels of ubiquitination are the key, it is easy to understand how Bsd2 can replace the need for Ear1/Ssh4 and Pib1, and why different cargo proteins may have slightly different requirements–depending, e.g. on the availability of lysine residues as well as the efficiency with which they interact with the various adaptors. It is also consistent with the observation that limiting the activity of arrestins and Bul proteins can slow the passage of transporters through endosomes, as well as their internalization.

It seems most likely that the additional ubiquitination occurs mainly in endosomes, as Bsd2 and Ear1/Ssh4 are membrane proteins that are themselves substrates for Rsp5 and are targeted to the vacuole apparently without reaching the plasma membrane (5). Furthermore, Pib1 is bound to membranes via a PX domain, which recognizes PI3P, which in turn is found on endosomes rather than at the cell surface (24). It is also likely that it is ubiquitination of the transporters themselves, rather than the endocytic machinery, that is required. This is borne out by the different requirements of different transporters, and by direct evidence for Ear1/Ssh4 dependent ubiquitination of the siderophore transporter Sit1 (5), and Bsd2-dependent ubiquitination of Smf1 (4). We have also observed that mutation of eight lysine residues in the third cytoplasmic loop of Smf1, which are distinct from the N-terminal lysines that undergo arrestin-mediated modification (12), slows delivery to the vacuole in *bsd2* cells (our unpublished observations), suggesting that these lysines may be sites of Pib1 and/or Ear1/Ssh4 dependent ubiquitination.

One obvious question is how these disparate proteins recognize their substrates. Ear1 and Ssh4 contain SPRY domains, which may mediate protein–protein interactions, but they appear to act on every protein tested, from the vacuolar enzyme Phm5, with a single transmembrane domain and a tiny cytoplasmic domain, to large multispanning transporters such as Fur4 and Smf1 (5). Pib1 has little other than its PX and RING domains. Bsd2 recognizes transmembrane sequences, though in the case of Smf1 recognition may be indirect, via Tre1/2 (4,25). It is possible that all the transporters whose modification depends on these proteins are specifically recognized by each of them, in a manner that depends on stress or ligand. An alternative, but not mutually exclusive explanation is that modification is controlled largely by location. As all the relevant proteins are ubiquitinated, including transporters and adaptors, they will all be associated with ESCRT complexes, and this juxtaposition may be enough in at least some cases to ensure ongoing modification of cargo molecules.

These considerations suggest a view of transporter regulation in which specificity is provided by arrestin interactions which promote initial internalization, and this is followed by relatively non-specific further ubiquitination in endosomes. This places great importance on the initial endocytic step, and it is worth considering what else might influence this. Notably, it has been shown that Can1, Tat2 and Fur4 are localized within small highly stable ergosterol-rich compartments in the plasma membrane whose formation depends on specific proteins, and that this slows their endocytosis by sequestering them from the sites at which this happens (32–34). In an *erg6* mutant, defective in ergosterol synthesis, the stable compartments do not form (32), and Tat2 is constitutively ubiquitinated and delivered to the multivesicular body pathway (23). Moreover, addition of arginine releases Can1 from the ergosterol-rich compartments and thus facilitates its internalization and degradation (32). Thus in an extreme case, ligand or stress could control the fate of a transporter simply by releasing it from an immobilized state, allowing relatively non-specific interactions with arrestins and other adaptors to promote its degradation. Such a mechanism could help to explain how regulation of Tat2 and Fur4 is maintained despite the multitude of adaptors that are able to mediate the destruction of these transporters–at least some of the adaptors would need only to recognize the proteins with moderate efficiency, not to distinguish subtle ligand-induced differences.

It seems that there are considerable differences in the way that the presence of individual transporters at the plasma membrane is controlled, though arrestins provide a common theme and may in some cases mediate specific regulatory mechanisms. Further exploration will no doubt reveal additional features, and may assign roles to the four arrestins whose functions are currently unknown. The arrestin-deficient strains we have developed should provide useful tools for future study.

## Materials and Methods

All yeast strains were derivatives of BY4741 (*MATa his3-*Δ*1 leu2-*Δ*0 met15-*Δ*0 ura3-*Δ*0*) or BY4742 (*MAT*α*his3-*Δ*1 leu2-*Δ*0 lys2-*Δ*0 ura3-*Δ*0*) and are listed in Table 3. Single deletion mutants in each mating type were obtained from the Euroscarf consortium. Double mutants were obtained by mating and sporulation (EN06-10) or by additional gene disruption. Multiple deletions were obtained by substituting the entire coding region of genes with a cassette containing the *Schizosaccharomyces pombe HIS5* gene flanked by loxP sites, then using transient expression of the cre protein from a plasmid to remove the *HIS5* marker.

**Table 3:** Yeast strains used in this study

Strain	Genotype
EN06	*rod1::G418 rog3::G418 his3 ura3 leu2*
EN07	*aly1::G418 aly2::G418 his3 ura3 leu2*
EN08	*ecm21::G418 csr2::G418 his3 ura3 leu2*
EN09	*ygr068c::G418 rim8::G418 his3 ura3 leu2*
EN10	*bul1::G418 bul2::G418 his3 ura3 leu2*
EN20	*bsd2::G418 pib1::natMX his3 ura3 leu2*
EN41	*ear1 ssh4 his3 ura3 leu2*
EN44	*pRSP5::natMX his3 ura3 leu2* (*rsp5* promoter insertion)
EN59	*ecm21::G418 csr2::G418 bsd2 rog3::natMX rod1 ygr068c aly2 aly1 ldb19 rim8 ylr392c::HIS his3 ura3 leu2*
EN60 (*9-arrestin*)	*ecm21::G418 csr2::G418 bsd2 rog3::natMX rod1 ygr068c aly2 aly1 ldb19 ylr392c::HIS his3 ura3 leu2*
EN63	*ecm21::G418 csr2::G418 bsd2 rog3::natMX rod1 ygr068c aly2 aly1 ldb19 ylr392c bul1::HIS his3 ura3 leu2*
EN65	*ear1 ssh4 bsd2::HIS his3 ura3 leu2*
EN67	*ecm21::G418 csr2::G418 bsd2 rog3::natMX rod1 ygr068c aly2 aly1 ldb19 ylr392c bul1 bul2::HIS his3 ura3 leu2*

Plasmids were based on the YCplac111 *CEN LEU2* or YCplac33 *CEN URA3* vector. Individual arrestins were expressed from their own promoters, GFP-Itr1, Fur4-GFP and Tat2-GFP from the *GAL1* promoter, and GFP-Smf1 and Hxt6-GFP from the *TPI* promoter. GFP-Itr1, Tat2-GFP and GFP-Smf1 were on *LEU2* plasmids, Hxt6-GFP and Fur4 on *URA3* plasmids. Co-expressed arrestins were on plasmids bearing the alternate marker (*URA3* or *LEU2*). Bsd2 was expressed from either its own promoter (with Fur4 and Hxt6) or the TPI promoter (with Tat2 and Itr1). Strains were grown in appropriate selective media to maintain the plasmids. Growth rates in Table 2 were determined in rich medium (1% yeast extract, 2% peptone, 2% glucose).

For fluorescence experiments cells were usually grown to exponential phase (OD_600_ less than 0.3). For Smf1, synthetic medium with glucose and appropriate supplements was used, and cadmium treatment was performed as described previously (12). For Itr1, cells were grown first in synthetic medium containing 2% raffinose and 0.2% glucose, then transferred to inositol-free yeast nitrogen base medium (MP Biomedicals) containing 2% raffinose and 0.2% glucose. Galactose (2%) was added and incubation continued for 4.5–5 h. Glucose (2%) was added to stop Itr1 expression, and endocytosis initiated by adding 60 µm myo-inositol. For Hxt6, cells were grown in YEP medium (1% yeast extract and 2% peptone) with 2% raffinose to an OD_600_ of approximately 1, then endocytosis induced either by adding 100 µg/mL cycloheximide, or by transferring to medium containing 0.17% yeast nitrogen base without ammonium sulphate or amino acids but with 5% glucose (17), or by additionally adding cycloheximide. For Fur4, cells were grown on normal medium lacking uracil but containing 2% raffinose and 0.2% glucose, transferred to medium containing 2% galactose for 3 h, then 2% glucose was added and the cells incubated for a further 30 min before induction of endocytosis with either 100 µg/mL cycloheximide or 40 µm uracil. For Tat2, the same protocol was used as for Fur4, except that cells were grown on synthetic medium lacking tryptophan and leucine, and endocytosis was induced by adding 1.5 mg/mL tryptophan.

Immunoblotting was performed on total cell extracts prepared using the alkaline lysis method (19). Rabbit anti-GFP antibodies were from Sigma-Aldrich, and anti-Pgk1 antibodies from Invitrogen.

Fluorescence imaging was performed on live cells in growth medium using a Zeiss LSM510 confocal microscope as previously described (12). Images were adjusted for brightness and contrast, inverted and in some cases blurred to reduce high-frequency noise using Adobe Photoshop.
